# Narcissism in Action: Perceptions, Team Dynamics, and Performance in Naturalistic Escape Room Settings

**DOI:** 10.3390/bs15111461

**Published:** 2025-10-27

**Authors:** Reece D. Bush-Evans, Claire M. Hart, Sylwia Z. Cisek, Liam P. Satchell, Constantine Sedikides

**Affiliations:** 1 School of Psychology, Bournemouth University, Poole House, Poole BH12 5BB, UK; 2 School of Psychology, Highfield Campus, University of Southampton, Southampton SO17 1BJ, UK; c.m.hart@soton.ac.uk (C.M.H.); s.z.cisek@soton.ac.uk (S.Z.C.); c.sedikides@soton.ac.uk (C.S.); 3 School of Psychology, Sport, and Health Sciences, University of Portsmouth, Portsmouth PO1 2UP, UK; liam.satchell@port.ac.uk; 4 Department of Psychology, University of Winchester, Winchester SO22 4NR, UK

**Keywords:** narcissism, team dynamics, cohesion, team conflict, team performance

## Abstract

We investigated narcissism in a naturalistic social context. Specifically, we examined how individuals high in admirative and rivalrous narcissism are perceived in team dynamics. Participants (*n* = 101) worked in small teams (*k* = 23 teams) during escape room-based tasks. Using a round-robin design, we observed alignment between self- and peer-ratings on interpersonal traits. Those high on admirative narcissism were perceived as confident but overestimated their likeability, whereas those high on rivalrous narcissism were perceived as aggressive and lazy. Teams characterized by high levels of rivalry exhibited reduced team cohesion, which in turn was associated with poorer team performance. There were no team-level effects for narcissistic admiration. The research advances understanding of admirative and rivalrous narcissism by simulating real-time teamwork in escape rooms.

## 1. Narcissism in Action: Perceptions, Team Dynamics, and Performance in Naturalistic Escape Room Settings

A team is the product of its parts, and as such, an individual’s personality can serve as the glue that holds a team together or the sledgehammer that breaks it apart. Effective teams rely on high-quality interactions among members, collective decision-making, and a willingness to prioritize team outcomes over individual interests (de la Torre-Ruiz et al., 2014; Morgan et al., 2021; Shaw-Ree, 2025). These processes foster team cohesion (Forsyth, 2021; Kwon, 2024), reduce conflict (Nunkoo & Sungkur, 2021; van Woerkom & Sanders, 2009), and enhance performance (Marlow et al., 2018; Zhu et al., 2020). Yet, when certain personality traits disrupt these dynamics, they can undermine a team’s ability to function effectively (Baysinger et al., 2025; Dillon et al., 2021). One such trait that may threaten the cooperative foundation on which successful teamwork depends is narcissism, characterized by grandiosity, an inflated sense of self-importance and entitlement, and a lack of empathy or concern for others (Sedikides, 2021; Thomaes et al., 2018).

Although previous research has examined how narcissism shapes team dynamics and performance (Grijalva et al., 2020; Lynch et al., 2021; Nevicka et al., 2023; Roberts et al., 2017), there is a notable scarcity of studies using naturalistic, time-pressured team tasks that allow for direct behavioral observation. We address this gap by using a commercial escape room as a realistic simulation of teamwork, requiring teams to communicate, problem-solve, and collaborate under pressure. We examine how narcissism influences team processes and performance within an escape room setting, offering insights into the ways personality drives team success or derails collective efforts under pressure.

### 1.1. Team Processes and Performance

Teams thrive when members trust one another (McNeese et al., 2021), share decision-making responsibilities (Imam & Zaheer, 2021), exchange task-relevant information (Mesmer-Magnus & DeChurch, 2009), communicate effectively (Brewer & Holmes, 2016; Zhou & Gorman, 2024), and draw on their diverse skills to achieve shared goals (Tseng et al., 2024). The quality of these interactions is shaped by members’ attributes and behaviors (Bell, 2007; Kozlowski & Bell, 2003), with productive teams characterized by open communication, equal participation, clear goals, and a shared vision (Hakanen & Soudunsaari, 2012; Hecht et al., 2023). These collaborative processes not only facilitate task completion but also influence key team processes, including cohesion, conflict management, and overall team performance (Downes et al., 2021; Kozlowski & Ilgen, 2006; Van Mierlo & Van Hooft, 2020).

Among these processes, team cohesion is considered one of the strongest predictors of team performance (Gächter et al., 2025; Wei et al., 2024). Cohesion refers to the shared bond and commitment that motivates team members to work together and remain united to reach a common outcome (Carron et al., 1998; Kozlowski & Ilgen, 2006). When team members feel connected, they are more likely to engage in cooperative behaviors (Liang et al., 2015), support one another (Hoegl & Proserpio, 2004), and persist in working towards shared objectives, even in the face of challenges (Alliger et al., 2015; López-Gajardo et al., 2022). High levels of cohesion are associated with greater team trust and satisfaction (DeOrtentiis et al., 2013; Fung, 2014), enhanced psychological well-being (Chen et al., 2009), a stronger sense of belonging within the team (Friedkin, 2004), and increased motivation among team members (Berengüí et al., 2021). In contrast, when cohesion is low, team members may withdraw or disengage, reducing collaboration and undermining the team’s potential for success (Grossman et al., 2021; Park et al., 2021). Although cohesive teams often demonstrate higher performance across a variety of contexts and team types (Braun et al., 2020; Franz et al., 2017; Tavoletti et al., 2025), conflict can nevertheless still arise (Cosier & Rose, 1977; Garner, 2021).

Conflict is natural. Teams will inevitably experience conflict (Adham, 2023; Santos et al., 2022). Broadly defined as an incompatibility between two or more opinions, principles, or values (Jehn & Mannix, 2001; Littlejohn & Domenici, 2001), team conflict arises when members disagree over goals (Korsgaard et al., 2008) or perceive their interests to be challenged by others (De Dreu & Gelfand, 2008). When teams fail to manage these differences constructively, conflict can escalate. High levels of team conflict have been linked to negative outcomes, including reduced team cohesion (Tekleab et al., 2009), lower creativity (Rong et al., 2019), increased bullying (Baillien et al., 2015), and impaired team performance (van Woerkom & van Engen, 2009).

Intragroup conflict can be conceptualized as relationship conflict and task conflict (Jehn, 1995). Relationship conflict involves interpersonal tensions, personality clashes, and feelings of animosity among team members (Jehn & Mannix, 2001; Pelled, 1996), and has been linked to reduced trust (Rispens et al., 2007) as well as poorer team creativity and performance (Farh et al., 2010). In contrast, task conflict includes disagreements over procedures and the allocation of resources within a team (Jehn, 1995). Although often associated with lower team member satisfaction (Hinds & Mortensen, 2005) and reduced performance (Puck & Pregernig, 2014), task conflict can also promote decision-making, broaden the knowledge pool, and stimulate creativity and innovation (Bang & Park, 2015; de Wit et al., 2013; Salcinovic et al., 2022).

Personality plays a critical role in shaping team processes and outcomes, including cohesion, conflict, and performance (Dillon et al., 2021; Hancock & Hill, 2022; O’Neill & Allen, 2011; Pérez-Luño et al., 2024). Given that individual differences emerge as important factors in how a team functions, it is essential to investigate specific traits that may either support or undermine effective teamwork. We focus on narcissism.

### 1.2. Narcissism in Teams

As mentioned above, narcissism is marked by excessive self-importance, lofty self-views, a continual desire for recognition, and diminished concern for others (Morf & Rhodewalt, 2001; Sedikides, 2021). Individuals high in narcissism perceive themselves as superior on agentic traits such as creativity, intelligence, and power (Macenczak et al., 2016; Zajenkowski et al., 2019; Zheng et al., 2025) and feel entitled to desirable outcomes (Queiri & Alhejji, 2025). They are overconfident in their abilities (Campbell et al., 2004), are eager to present themselves favorably (Vanhoffelen et al., 2024), and boast (Grijalva & Zhang, 2015). Unsurprisingly, narcissists do not make the best team members.

Within teams, narcissists crave credit for their own contributions (Liu et al., 2022), dominate discussions (Choi & Phan, 2022), dismiss advice from others (Kausel et al., 2015), and claim credit for collective success while blaming others for failures (Campbell et al., 2000). Their self-serving behaviors, such as withholding information (Lakey et al., 2008), devaluing teammates (Locke, 2009), and prioritizing personal gain over collective interests (Arthur et al., 2011; Campbell et al., 2005), often undermine collaboration and fuel conflict (Lynch et al., 2021). Driven by a zero-sum mindset, narcissists frequently disregard the feelings of their team members (Vonk et al., 2013), react aggressively when they feel undervalued or fail to get their own way (Gardner & Pierce, 2011), and are less likely to support goals or decisions proposed by others (Giambatista & Hoover, 2018), further disrupting processes essential for effective teamwork.

To maintain a grandiose self-image, narcissists engage in various cognitive and interpersonal strategies, including self-promotion (Moon et al., 2016) and status seeking (Zeigler-Hill et al., 2018), often at the expense of others. Given their strong desire to get ahead, the presence of narcissism within teams may influence team processes such as cohesion, conflict, and performance. Yet, despite widespread recognition of the social consequences of narcissism, including pursuing unrealistic projects (Wales et al., 2013) and exploiting others for their own gains (Khoo & Burch, 2008), implications for team processes remain underexplored.

Although early research treated grandiose narcissism as a single construct, more recent theory distinguishes two interrelated but distinct dimensions: narcissistic admiration and narcissistic rivalry. Both serve the overarching goal of maintaining a grandiose self-view, but they do so through different strategies. Admiration reflects assertive self-enhancement (e.g., charm, charisma, striving for uniqueness), whereas rivalry reflects antagonistic self-protection (e.g., hostility, devaluing others). Their positive correlation explains why unidimensional measures produced high internal consistency in past work, yet considering them separately has revealed divergent social and interpersonal consequences (Back et al., 2013; Leckelt et al., 2015).

Despite growing evidence that narcissistic admiration and rivalry are associated with intra- and interpersonal shortcomings that are considered detrimental in team contexts (Back et al., 2013; Grijalva et al., 2020; Nevicka et al., 2011), research exploring how these forms specifically relate to team processes and performance is limited. We apply the Narcissistic Admiration and Rivalry Concept (NARC; Back et al., 2013) to test how teams with higher levels of narcissism experience increased conflict and reduced cohesion, and how these processes, in turn, affect overall team performance.

### 1.3. The Role of Narcissistic Admiration and Rivalry in Shaping Team Processes and Performance

Narcissistic rivalry reflects antagonistic self-protection and is characterized by striving for supremacy, devaluing others, and engaging in aggressive behaviors (Back et al., 2013; Kirk et al., 2025). Driven by a fear of failure, individuals high in rivalry defend their perceived superiority when threatened, often culminating in hostility, rejection, and distrust from others (Back, 2018; Lange et al., 2016). Within teams, those high in rivalry may withhold information, dismiss others’ contributions, filter out feedback that challenges their self-beliefs, derogate team members during discussions, and aggressively push their own ideas. Indeed, individuals high in rivalry often exhibit a lack of regard for others that can undermine leader effectiveness (Lynch & Benson, 2023), adopt dysfunctional conflict patterns within teams (Boulter et al., 2022), and promote competitive rather than cooperative behaviors in team settings (Lynch et al., 2021). Additionally, narcissism may erode task cohesion (Boulter et al., 2021), as narcissists perceive disagreements about abilities or competence as potential self-threats, prompting aggressive responses toward those who challenge them. As narcissistic rivalry is linked to knowledge hiding in teams (Yang et al., 2025) and a stronger desire to abandon a team during tasks (Benson et al., 2018), we offer the following hypothesis:

**Hypothesis** **1.** *At the team level, higher narcissistic rivalry is negatively associated with performance by undermining cohesion and fostering greater conflict.*

Narcissistic admiration reflects assertive self-enhancement, expressed through striving for uniqueness, grandiose fantasies, and charm, and is driven by a desire for success and acclaim (Back, 2018; Back et al., 2013). These proclivities often engender favorable social outcomes, such as empowerment and leadership opportunities (Härtel et al., 2021; Helfrich & Dietl, 2019; Nevicka & Sedikides, 2021), which may reinforce a grandiose self-view. However, in team contexts, the pursuit of recognition may have mixed effects. On the one hand, the desire for personal success may lead narcissists to exploit the team, sparking conflict and undermining team performance. On the other hand, their agentic behaviors and desire for admiration may leave positive impressions on team members, especially in early interactions, temporarily boosting team processes and performance. These patterns are supported by findings that narcissists typically leverage team resources for self-interest (Campbell et al., 2005) and create good first impressions (Leckelt et al., 2015). Given that individuals high in narcissistic admiration are often socially skilled (Eddy, 2023), it is possible that they strategically help others to get ahead or hide their self-serving motives (Konrath et al., 2016). Consequently, they may be viewed more positively by their team members (albeit initially) and gel with their team members, at least until their motives become apparent over time (Leckelt et al., 2015). Specifically, we hypothesize:

**Hypothesis** **2.** *At the team level, higher narcissistic admiration is associated with improved performance by boosting cohesion and reducing conflict, particularly in short-term tasks like an escape room.*

### 1.4. Research Overview

Many phenomena in social science are interpersonal, requiring analysis that goes beyond individuals in isolation to consider how people perceive, respond to, and affect each other within social contexts (Back & Kenny, 2010). Traditional approaches often overlook these relational dynamics, yet in teams, individuals are simultaneously perceivers and targets, influencing and being influenced by others around them. To understand how narcissism shapes behaviors within teams, it is crucial to examine relational and team-level processes, capturing how individuals interact, how they are perceived by team members, and how these perceptions may influence team processes and performance.

The social relations model (Back & Kenny, 2010; Kenny, 1988) offers a framework to disentangle these complexities by capturing perceiver effects (how an individual generally perceives others), target effects (how an individual is generally perceived by others), and relationship effects (the unique perception one individual holds toward another, beyond perceiver and target effects). By using a round-robin design, where each team member interacts with or rates every other member, the social relations model allows the systematic study of interpersonal dynamics in teams. This approach has been used to examine perceived student performance (Horn et al., 1998), teamwork perceptions (LeDoux et al., 2011), and the association between interpersonal attraction and personality (Küfner et al., 2013). The social relations model has also advanced narcissism research, highlighting peer-rated leadership (Ong et al., 2016), awareness of social status over time (Carlson & DesJardins, 2015), perceptions of popularity (Rentzsch & Gebauer, 2019), upward-status disagreement in design teams (Xu & Benson, 2024), as well as admiration and likeability in team contexts (Grosz et al., 2024).

To capture the social consequences of narcissism within teams, we used a commercial escape room as a naturalistic, high-pressure team task requiring communication, collaboration, and collective problem-solving. Escape rooms are interactive and team-based games in which players are ‘locked’ in a room and must solve a series of puzzles within a set time limit to escape (Nicholson, 2015). Escape rooms can foster team cohesion (Cohen et al., 2021; Zhang et al., 2018), enhance problem-based learning (Nelson et al., 2017), as well as provide the context for measuring collaboration skills (Pan et al., 2017) and assessing teamwork under time pressure (Quek et al., 2024; Stasiak, 2019). Given their inherently competitive and time-sensitive character, escape rooms provide narcissists with opportunities to showcase their perceived competence and seek recognition. This property renders escape rooms an optimal setting to explore how narcissism contributes to team processes and performance when one is driven to stand out while still depending on others for achieving a shared goal. We advance the following hypotheses:

**Hypothesis** **3.** *At the relational level, individuals high in narcissistic rivalry are consistently viewed negatively by team members (e.g., arrogance, aggression) across both time points.*

**Hypothesis** **4.** *At the relational level, individuals high in narcissistic admiration are viewed more positively at the start of the team task (e.g., likeable, confident), but these impressions decline over time.*

The present study makes three contributions. First, we advance research on narcissism in team contexts by distinguishing between narcissistic admiration and rivalry, and testing their effects on cohesion, conflict, and performance in a naturalistic, time-pressured task. Second, we integrate both subjective and objective performance outcomes, offering a more comprehensive assessment of how personality influences team functioning. Third, by employing a round-robin design, we capture interpersonal perceptions within teams, allowing us to examine not only team-level effects but also how individuals high in narcissism are perceived by peers over time. Together, these contributions provide a more nuanced understanding of narcissism’s role in shaping team dynamics and outcomes.

## 2. Method

### 2.1. Participants

We recruited participants via social media platforms (i.e., Facebook, X) and in-person through leaflet distribution and public interaction “to take part in a study on personality and teamwork.” Self-selection introduces sampling bias but aligns with common practices in team research (Fischer et al., 2023). Eligibility criteria required participants to be at least 18 years old and available on the scheduled study dates. Our intended sample size rationale was based on what was practically feasible for our project (for more on practical considerations for sample sizes, see Lakens, 2022). The sample included 101 participants (59 women, 42 men), who ranged in age from 18 to 64 years (*M* = 29.87, *SD* = 10.19). Most of them were White British (78.2%), followed by other White backgrounds (9.9%), Indian (5.0%), White and Black Caribbean (3.0%), and other ethnic backgrounds (3.9%).

Nearly half of the participants were employed full-time (49.5%) and had completed higher education (44.6%). Participants worked across sectors including education (13.9%), health and social care (10.9%), retail (10.9%), and software (8.9%). Many held management positions (32.7%) and worked in team-oriented roles (50.5%). Most of them (62.4%) had never played an escape room, whereas those with prior experience (37.6%) had typically played only one (63.6%) or three (21.2%) games.

The study included 9878 individual ratings: 101 participants providing self-ratings and other-ratings on 11 measures across two time points. The round-robin design allows for the analysis of interpersonal perceptions while controlling for rater-specific error. To assess the sensitivity of team-level tests, we conducted a sensitivity analysis (*a* = .05, power = .80) for team-level bivariate associations given *k* = 23 teams. The analysis indicated a smallest detectable correlation of *r* ≈ .56 (equivalent to Cohen’s f^2^ ≈ .45 (a large effect). Consequently, while the study was sufficiently powered to detect medium-to-large team-level effects, it had limited sensitivity to detect small or small-to-medium effects at the team level. By contrast, the round-robin relational component produced 9878 individual ratings and, together with social-relations modelling (partitioning perceiver/target/relationship variance), provided substantially greater precision for detecting small-to-moderate effects in interpersonal perceptions.

### 2.2. Procedure

The study, approved by the Ethics Committee of the School of Psychology at the University of Southampton (ERGO 31132), was advertised as a jungle-themed escape room game. The advertisement included a link to the Participant Information Sheet, which outlined the study structure: An online pre-test questionnaire followed by an escape room session with additional questionnaires. After completing the pre-test questionnaire, participants provided contact details and availability for the escape room session and received an invite code for a discounted future escape room game at the venue.

For the escape room session, participants were assigned to teams based on availability and invited to the escape room venue (*k* = 23 teams, Range = 4–5 members, *M_size_* = 4.45). Some team members may have known each other due to similar availability^1^. Upon arrival, participants received study information before sitting around in a circle with their team.

Participants completed a 5-min icebreaker (i.e., getting-to-know-you task) based on the relationship closeness induction task (Sedikides et al., 1999). This involved answering and sharing responses to seven introductory questions (e.g., “What is your name?”, “Where are you from?”, “What is something you have always wanted to do but probably never will be able to do?”). Next, participants were given a clipboard with the first in-person questionnaire (Time 1: Post-Icebreaker), where they rated themselves and each teammate on interpersonal and team-related attributes (in a round-robin fashion). Venue staff then provided instructions and a narrative overview of the escape room game. Participants received a walkie-talkie for game-related communication only (e.g., requesting hints, receiving instructions, communicating with the researcher if needed). Finally, they were led to the escape room entrance and informed that they had 60 min to complete the tasks and escape.

The jungle-themed escape venue featured three connected rooms requiring teamwork to solve puzzles (Room 1: Puzzle room with four tasks; Room 2: Jungle room with four tasks; Room 3: Cave room with three tasks). Tasks included decoding a fictional language, locating hidden artefacts, and matching patterns to unlock sequences. Teams needed to complete all tasks to proceed to the next room and finish the game, with some teams remaining in a room until the end if they could not solve all tasks.

Games were recorded using venue cameras while the researcher observed live, noting any issues. After 60-min, the researcher ended the game for teams still playing and provided a brief game summary (e.g., pass or fail). Participants then returned to the icebreaker room to complete the second questionnaire (Time 2: Post-Escape Room), which included the same interpersonal ratings (now based on in-game interactions) along with additional items on team processes and performance. Finally, participants exited the venue. Although participants had only just met at Time 1, these early assessments are important in time-pressured tasks where individuals must quickly form impressions to guide interactions. Consistent with theories on thin-slice judgment, such immediate evaluations possess adaptive value and, even at zero acquaintance, have been shown to reliably predict interpersonal outcomes (Ambady et al., 1995; Rau et al., 2022).

### 2.3. Measures

#### 2.3.1. Pre-Test Questionnaire Measures

##### Narcissistic Admiration and Rivalry

We measured this construct with the 18-item Narcissistic Admiration and Rivalry Questionnaire (Back et al., 2013). It includes nine items assessing narcissistic admiration (e.g., “I manage to be the center of attention with my outstanding contributions”) and nine assessing narcissistic rivalry (e.g., “I react annoyed if another person steals the show from me”; 1 = *strongly disagree*, 8 = *strongly agree*). We averaged scores for each form, with higher scores indicating greater levels of narcissistic admiration (Range = 1.44–7.77, *M* = 4.27, *SD* = 1.26, *a* = .85) and narcissistic rivalry (Range = 1.00–5.44, *M* = 2.66, *SD* = 1.01, *a* = .78). To examine team-level effects, we aggregated individuals’ scores within each team, computing team-level averages for narcissistic admiration (Range = 2.67–5.31, *M* = 4.27, *SD* = .58) and narcissistic rivalry (Range = 1.92–3.78, *M* = 2.66, *SD* = .49). This decision was guided by prior work on team personality composition (e.g., Boulter et al., 2022; Lynch et al., 2021; Schmid et al., 2021).

#### 2.3.2. Impression Management

We measured this construct with the 8-item Impression Management subscale of the BIDR-16 (C. M. Hart et al., 2015). This subscale captures deliberate efforts to please others, with items such as “I sometimes tell lies if I have to” and “I don’t gossip about other people’s business” (1 = *strongly disagree*, 8 = *strongly agree*). We reverse-scored four items. We included this scale as a covariate in analyses (Range = 2.00–7.75, *M* = 5.04, *SD* = 1.25, *a* = .74) to account for the influence of socially desirable responding, which can systematically bias self-report data (Alexander et al., 2025). Accounting for this factor is especially important given narcissists’ propensity for strategic self-presentation and heightened self-regard (W. Hart et al., 2017).

#### 2.3.3. Escape Room Session Measures

##### Interpersonal Perceptions

Participants rated themselves and each team member on an 11-item interpersonal perception measure that we developed for this study, based in part on validated scales (Campbell et al., 2005; Paulhus, 1998). Sample items are “This person is supportive,” “This person is likeable,” “This person is trustworthy,” “This person is aggressive,” and “This person is empathic” (1 = *not at all*, 8 = *extremely*). Participants completed ratings at two time points: Time 1 (Post-Icebreaker) and Time 2 (Post-Escape Room).

##### Team Cohesion and Collaboration

We assessed perceived team cohesion and collaboration across tasks with the 9-item Group Cohesion Evaluation Questionnaire (Glass & Benshoff, 2002). Sample items are “We enjoyed helping each other” and “I felt confident working with my team on challenging tasks “(1 = *strongly disagree*, 8 = *strongly agree*). We averaged scores, with higher scores indicating greater perceived team cohesion and collaboration (Range = 2.00–8.00, *M* = 6.05, *SD* = 1.33, *a* = .95). We calculated team-level cohesion by aggregating individual scores within each team (Range = 4.44–7.20, *M* = 6.05, *SD* = .80). The intraclass correlation coefficients (ICC) indicated that 18% of the variance was attributable to team membership (ICC[1] = .18), with a moderate reliability for the aggregated team means (ICC[2] = .50), supporting aggregation to the team level (Bliese, 2000; LeBreton & Senter, 2007).

##### Team Conflict

We assessed perceived team conflict with the 9-item Intragroup Conflict Scale (Jehn, 1992). Sample items are “How much anger was there among team members?” and “Was decision-making problematic in your team?” (1 = *none/not at all*, 8 = *a lot/very much so*). We averaged scores, with higher scores indicating greater perceived team conflict (*Range* = 1.00–7.11, *M* = 2.65, *SD* = 1.37, *a* = .92). Team-level conflict was calculated by aggregating individual scores within each team (Range = 1.28–4.29, *M* = 2.65, *SD* = .86). Of the variance, 22% was attributable to team membership (ICC[1] = .22), with a moderate reliability for the aggregated team means (ICC[2] = .56). Due to the high intercorrelation among relationship conflict and task conflict subscales (*r* = .78), we used only the total conflict score to prioritize parsimony and reduce redundancy.

##### Team Performance

We assessed team performance using both subjective and objective indicators. We measured subjective team performance with an 8-item scale that we constructed for the purpose of this study. Participants rated statements such as “My team overcame difficulties to get the job done,” “My team was successful at solving problems,” “My team was productive at completing tasks most of the time,” and “My team produced good results” (1 = *strongly disagree*, 8 = *strongly agree*). Three items were reverse scored (“My team often struggled to solve the puzzles,” “My team failed to complete tasks effectively,” and “My team failed to complete the task on time”). We averaged scores, with higher scores reflecting greater perceived team performance (Range = 1.63–8.00, *M* = 5.38, *SD* = 1.46, *a* = .91). To examine team-level effects, we aggregated individual scores within each team to compute team-level subjective performance (Range = 2.63–7.34, *M* = 5.38, *SD* = 1.64). The reliability of team means was excellent (ICC[1] = .74 and ICC[2] = .93).

We assessed objective team performance as the total number of game rooms completed by each team during the escape room task (Range = 0–3, *M* = 1.83, *SD* = 1.12). Specifically, 17.4% (four) of teams completed zero rooms, 21.7% (five) completed one room, 21.7% (five) completed two rooms, and 39.1% (nine) completed all three rooms.

### 2.4. Data Analysis

We were interested in team- and relational-level influences upon team processes (cohesion, conflict) and team performance in a naturalistic team setting. For team-level analyses, we conducted multiple mediation models (PROCESS Model 4, Hayes, 2022) in SPSS version 29.0.2.0 (IBM Corp, 2023) to examine how team-level narcissistic admiration and rivalry predicted team processes and performance.

An independent samples *t*-test identified significant gender differences in narcissistic admiration (*p* = .016, *d* = .49) and narcissistic rivalry (*p* = .009, *d* = .55). Consistent with prior research (Back et al., 2013; Leckelt et al., 2015), men reported higher levels of narcissistic admiration (*M* = 4.63, *SD* = 1.29) and narcissistic rivalry (*M* = 2.97, *SD* = .84) than women (*M* = 4.01, *SD* = 1.18 and *M* = 2.44, *SD* = 1.06, respectively). Thus, we included gender along with impression management and the alternative narcissism form as covariates in all models.

For relational-level analyses, we used a round-robin design involving interpersonal perceptions at Time 1 (Post-Icebreaker) and Time 2 (Post-Escape Room). We applied the social relations model (Kenny, 1994) to account for interdependence within team ratings by partitioning variance in perceptions (e.g., likeability) into perceiver, target, and relationship effects. We conducted analyses via the TripleR package (Version 1.5.4; Schönbrodt et al., 2012) in R (Version 4.5.1; R Core Team, 2025).

We carried out univariate round-robin analyses for each interpersonal perception. Via partial correlations, we assessed assumed similarity (correlations between self-ratings and perceiver effects) and self-other agreement (correlations between self-ratings and target effects). Via additional partial correlations, we examined associations between target effects and narcissism while controlling for team membership. We used target effects, as they reflect team-level agreement on how individuals are perceived, independent of perceiver or relationship biases. Following Kwon (2024), we computed self-enhancement indices for each interpersonal perception to obtain unbiased estimates of the extent to which individuals overestimate- or underestimate how they are perceived by their team.

## 3. Results

We provide descriptive statistics and reliability estimates (Cronbach’s alpha) in Table 1. Normality checks confirmed that assumptions for the planned analyses were met. We present correlations among study variables in Table 2. As expected, cohesion, conflict, and subjective performance were strongly correlated. Subjective and objective performance also showed a high correlation (*r* = .90), which likely reflects the salience of the escape room outcome (i.e., success vs. failure) as a shared reference point for both observed and self-reported performance.

To examine whether the final escape-room outcome influenced team-level ratings, we compared successful (*n* = 9) and unsuccessful teams (*n* = 14) on team-level cohesion and conflict. Teams that succeeded reported somewhat higher cohesion (*M* = 6.35, *SD* = .59) than teams that failed (*M* = 5.84, *SD* = .91), *t*(21) = −1.50, *p* = .147. Likewise, successful teams reported lower conflict (*M* = 2.26, *SD* = .84) than unsuccessful teams (*M* = 2.88, *SD* = .83), *t*(21) = 1.73, *p* = .098. Although these differences trended in the expected direction, they were not statistically significant, suggesting that the final outcome did not substantially bias ratings of team cohesion or conflict.

To test the proposed pathways, we conducted multiple mediation analyses using PROCESS Model 4 (Hayes, 2022). The analyses examined the effects of team-level narcissism on team processes (e.g., team conflict, team cohesion) and team performance through multiple regression pathways (Table 3). All models employed 5000 bootstrap samples and included gender, impression management, and the alternative narcissism form as covariates.^2^

### 3.1. Team-Level Narcissism and Performance: Mediation by Team Processes

**Figure 1:**
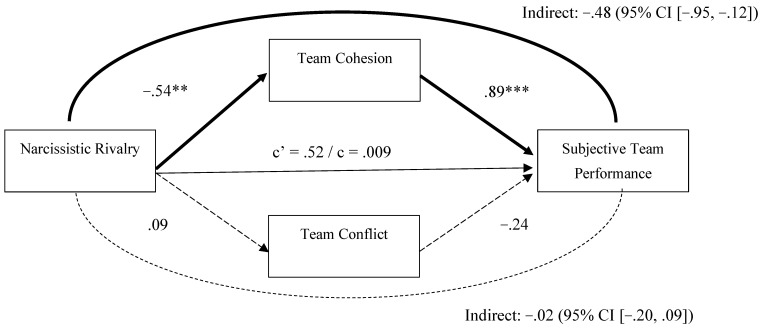
Mediation Analysis (PROCESS Model 4; Hayes, 2022) for Narcissistic Rivalry as the Predictor of Subjective Team Performance. *Note.* In this mediation analysis, covariates including gender, impression management, and narcissistic admiration were included, but their effects are not shown in the figure above for clarity. Significant and non-significant regression paths are indicated using regular (non-dashed) and dashed arrows, respectively. The path coefficients are unstandardized regression coefficients. *** *p* < .001. ** *p* < .01.

We illustrate in Figure 1 the direct and indirect associations between team-level narcissistic rivalry and subjective team performance via team cohesion and team conflict. Teams with high levels of narcissistic rivalry reported low team cohesion, which in turn was significantly associated with reduced subjective team performance. We found no significant indirect effects for team-level conflict on subjective team performance. The total effect of team-level narcissistic rivalry on subjective team performance was not significant, nor was the direct effect when controlling for the mediators. The model explained 37% of the variance in subjective team performance (R^2^ = .37).

**Figure 2:**
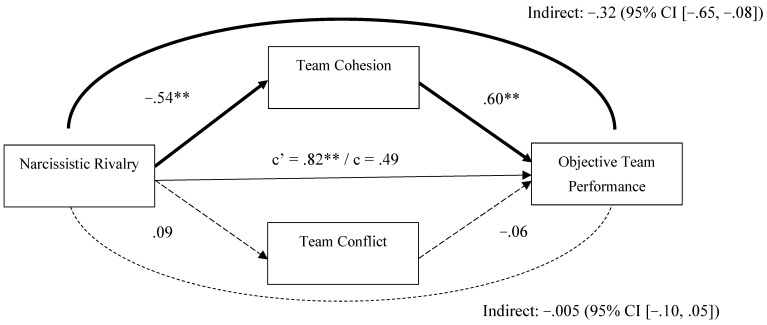
Mediation Analysis (PROCESS Model 4; Hayes, 2022) for Narcissistic Rivalry as the Predictor of Objective Team Performance. *Note.* In this mediation analysis, covariates including gender, impression management, and narcissistic admiration were included, but their effects are not shown in the figure above for clarity. Significant and non-significant regression paths are indicated using regular (non-dashed) and dashed arrows, respectively. The path coefficients are unstandardized regression coefficients. ** *p* < .01.

Another model using objective team performance as the outcome (Figure 2) showed a similar pattern. Teams with high levels of narcissistic rivalry reported low team cohesion, which in turn was significantly associated with reduced objective team performance. Although the total effect was not significant, the direct effect became significant when accounting for the mediators, suggesting a potential suppression effect in which the indirect and direct pathways operate in opposing directions. The model explained 35% of the variance in objective team performance (R^2^ = .35).

**Figure 3:**
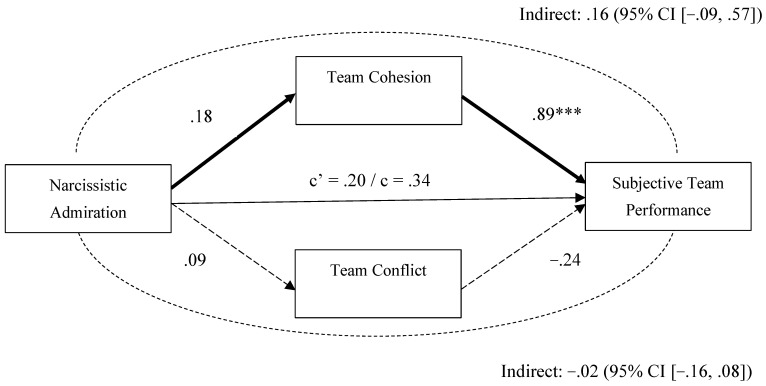
Mediation Analysis (PROCESS Model 4; Hayes, 2022) for Narcissistic Admiration as the Predictor of Subjective Team Performance. *Note.* In this mediation analysis, covariates including gender, impression management, and narcissistic rivalry were included, but their effects are not shown in the figure above for clarity. Significant and non-significant regression paths are indicated using regular (non-dashed) and dashed arrows, respectively. The path coefficients are unstandardized regression coefficients. *** *p* < .001.

**Figure 4:**
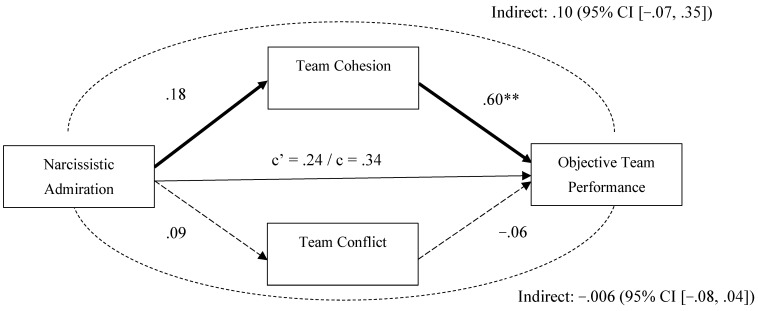
Mediation Analysis (PROCESS Model 4; Hayes, 2022) for Narcissistic Admiration as the Predictor of Objective Team Performance. *Note.* In this mediation analysis, covariates including gender, impression management, and narcissistic rivalry were included, but their effects are not shown in the figure above for clarity. Significant and non-significant regression paths are indicated using regular (non-dashed) and dashed arrows, respectively. The path coefficients are unstandardized regression coefficients. ** *p* < .01.

We illustrate in Figure 3 the direct and indirect associations between team-level narcissistic admiration and subjective team performance via team cohesion and team conflict. We observed no significant indirect, direct, or total effects. Another model using objective team performance as the outcome (Figure 4) showed a similar pattern with no significant indirect, direct, or total effects. These results indicate that the indirect pathway through reduced team cohesion is the key mechanism linking team-level narcissistic rivalry with team performance outcomes.

### 3.2. Social Relations Modelling of Interpersonal Perceptions

We summarize perceiver and target effects on interpersonal perceptions in Table 4. We found significant perceiver variance (*p*s < .01) across all interpersonal perceptions at both time points except for “good leader” at Time 1, indicating consistent individual differences in how participants rated their teammates. Also, we observed significant target variance (*p*s < .05) for “confident,” “supportive,” “arrogant,” “creative,” and “hardworking” at both time points, suggesting stable differences in how individuals were perceived by others. At Time 2, we found additional target variance for “aggressive,” “likeable,” “empathic,” and “good leader,” indicating that these perceptions develop through team interactions. No significant target effects emerged for “competent” or “trustworthy”.

We also examined assumed similarity (self-ratings correlated with perceiver effects) and self-other agreement (self-ratings correlated with target effects) to assess alignment between self-perceptions and team perceptions (Table 5). At Time 1, participants who rated themselves as arrogant, competent, aggressive, likeable, creative, or hardworking rated others similarly (*r*s > .23, *p*s < .05). At Time 2, these associations extended to confidence, supportiveness, trustworthiness, and empathy (*r*s > .29, *p*s < .05). For self-other agreement, participants who saw themselves as confident, likeable, empathic, or as good leaders at Time 1 (*r*s > .24, *p*s < .05), and as competent, empathic, or good leaders at Time 2 (*r*s > .26, *p*s < .05), were perceived as such by their teammates. No other significant effects emerged.

### 3.3. Narcissism and Interpersonal Perceptions: Partial Correlations

To examine associations between narcissism and interpersonal perceptions, we calculated partial correlations between narcissistic admiration and rivalry and the target effects of interpersonal perceptions, while controlling for team membership. Additionally, we computed self-enhancement indices to assess the extent to which participants underestimated or overestimated how they were perceived by their team, with negative scores indicating underestimation and positive scores indicating overestimation. We present correlations in Table 6.

Narcissistic admiration was associated with higher self-rated confidence (*r*s > .31, *p*s < .01) and peer-rated confidence (*r*s > .22, *p*s < .05) at both time points. It was also linked to being perceived as more arrogant (*r* = .28, *p* = .01) and less hardworking (*r* = −.24, *p* = .03) at Time 1, and more arrogant (*r* = .23, *p* = .04) and less empathic (*r* = −.36, *p* < .01) at Time 2. Participants high in narcissistic admiration also perceived themselves as more likeable at both time points (*r*s > .36, *p*s < .01), overestimated how likeable they were seen by others at Time 2 (*r* = .24, *p* = .03), and viewed themselves as better leaders at both time points (*r*s > .24, *p* < .03).

Narcissistic rivalry was associated with perceiving oneself and being perceived by others as more aggressive (*r* = .25, *p* = .03; *r* = .22, *p* = .05, respectively) and less hardworking (*r* = −.25, *p* = .03; *r* = −.25, *p* = .02, respectively) at Time 1. At Time 2, individuals high in narcissistic rivalry viewed themselves as more arrogant (*r* = .24, *p* = .04), aggressive (*r* = .23, *p* = .04), and less trustworthy (*r* = −.23, *p* = .04), and underestimated how likeable they were perceived by others (*r* = −.28, *p* = .01).

## 4. Discussion

We investigated narcissism within a naturalistic team setting, examining its influence at both the team level and relational level on team processes (cohesion and conflict) and team performance. The findings revealed that team-level narcissistic rivalry indirectly influenced team performance through its negative effect on team cohesion, supporting Hypothesis 1. In contrast, narcissistic admiration did not exhibit significant effects at the team level, providing no support for Hypothesis 2. At the relational level, narcissistic admiration and rivalry influenced how individuals perceived themselves and were viewed by others in the team, partially supporting Hypotheses 3 and 4. These findings highlight the interpersonal nuances of narcissism within team settings.

As expected, team-level narcissistic rivalry indirectly impaired team performance through its detrimental effect on team cohesion. Teams with higher levels of narcissistic rivalry, characterized by antagonism, hostility, and defensive self-protection (Back et al., 2013; Gauglitz et al., 2022; Lange et al., 2016), reported lower cohesion, which in turn was associated with poorer subjective and objective performance. This finding aligns with research indicating that narcissism can undermine team cohesion (Boulter et al., 2021) and that individuals high in narcissistic rivalry distance themselves from team members (Benson et al., 2018) and prefer competitive over cooperative environments (Lynch et al., 2021). Given the critical role of cohesion in team performance (Gächter et al., 2025; Wei et al., 2024), this finding contributes to the growing narcissism-performance literature (Harms et al., 2023; Lynch & Benson, 2023; Roberts et al., 2019) and highlights the potential for individuals high in narcissistic rivalry to subvert the collective bond required to keep teams productive.

Contrary to expectations, narcissistic rivalry was unassociated with increased team conflict. This pattern contrasts with prior research linking rivalry to heightened interpersonal conflict (De Clercq et al., 2022), dysfunctional team processes (Lynch et al., 2021), elevated conflict levels that impair performance (Harms et al., 2023), and ingroup devaluation (Xu & Benson, 2024). Various explanations may account for this null pattern. First, rivalry’s interpersonal costs often escalate over longer periods (Back et al., 2013; Leckelt et al., 2015), and the brief 60-min task may not have allowed these dynamics to emerge fully. Second, individuals high in rivalry may have engaged in indirect antagonism (e.g., social withdrawal, withholding task-relevant information; Lakey et al., 2008) rather than overt confrontation, making rivalry-driven behaviors less visible. Third, as teams were observed live by the researcher, participants may have suppressed arrogant-aggressive behaviors to avoid social disapproval, similar to patterns seen in short getting-acquainted contexts (Back, 2018; Grapsas et al., 2019; Küfner et al., 2013). Finally, in a time-constrained setting, avoiding conflict may have been viewed as a practical strategy to complete the task effectively. This explanation is congruent with research showing that individuals high in narcissism often excel in decision-making tasks by prioritizing outcomes over process concerns (Byrne & Worthy, 2013). Taken together, although rivalry can be linked to decreased cohesion, it may not always translate into overt conflict in short-term team settings.

Narcissistic admiration at the team level did not predict cohesion, conflict, or performance, providing no support for Hypothesis 1. Whereas narcissistic admiration includes traits such as charm, assertiveness, and self-confidence (Back et al., 2013), which are often seen as beneficial in team settings (Fransen et al., 2015; Pearsall & Ellis, 2006), these did not manifest as measurable shifts in team processes when aggregated at the team level. This possibility concurs with research indicating that, although individuals high in admiration often excel in self-presentation and are perceived as effective leaders early in interactions (Back et al., 2018; Härtel et al., 2021), these effects may not persist over time (Grijalva et al., 2020) or shape deeper team processes such as cohesion and conflict (Kessler et al., 2013). Additionally, admiration’s potential benefits within teams may be more pronounced in contexts where individuals can use their charisma and self-presentation skills, such as formal leadership roles, sales pitches, or public presentations (Grijalva et al., 2015). These settings often provide opportunities for visibility and influence. In contrast, time-pressured environments like escape rooms promote more distributed leadership, limiting the influence of individual dominance or impression management.

At the relational level, we observed individual differences in how team members rated and were rated on various interpersonal attributes across time, with traits like confidence, supportiveness, arrogance, creativity, and hard work being consistently salient. Over time, additional traits such as aggression, likeability, empathy, and perceived leadership also showed interindividual differences, suggesting that these attributes may develop through team interaction (Delice et al., 2019; Kozlowski & Ilgen, 2006). Consistent with research on rating bias (Jawahar, 2001; LeDoux et al., 2011; Sedikides et al., 2021), people rated others similarly to how they saw themselves (assimilation effects), and self-perceptions often aligned with how others viewed them (consensus effects). This highlights how both individual biases and the team context influence interpersonal perceptions in teams, particularly in intense collaborative environments like an escape room.

Further, the relational-level analyses indicated that individuals high in admiration perceive themselves as confident and likeable, and are perceived by others as such, but are simultaneously seen as more arrogant and less empathic over time. This pattern aligns with the possibility that narcissism often brings positive social outcomes in the short-term but less so in the long-term (Campbell & Campbell, 2009). Indeed, admiration influences early social interactions, resulting in being liked and positively regarded (Back et al., 2013; Leckelt et al., 2015; Szabó et al., 2024). However, as interactions evolve, the self-enhancement and confidence characteristics of admiration may be perceived as less favorable (Back et al., 2013; Carlson et al., 2011), potentially undermining relational trust within teams.

Individuals high in narcissistic admiration rated themselves as better leaders at both time points and overestimated how likeable they were perceived by others during the team task. Although narcissists may have awareness of their social reputation (Carlson, 2012), they are also motivated to maintain a positive self-image and interpret feedback in a self-serving manner (Morf & Rhodewalt, 2001). This motivation often culminates in exaggerated belief in their abilities and an inflated sense of social value (Carlson & DesJardins, 2015). In line with this reasoning, narcissists overrate their leadership abilities compared to peer evaluations (Paulhus, 1998) and regard themselves as more likeable than others regarded them to be (Clifton et al., 2009). These patterns align with the current findings, suggesting that individuals high in narcissistic admiration maintain overly positive and inflated perceptions of their leadership and likeability within team settings, irrespective of team processes and performance.

For narcissistic rivalry, the analyses revealed associations with being perceived by others as more aggressive and arrogant, and as less hardworking and trustworthy. Additionally, individuals high in rivalry underestimated how likeable they were perceived to be, indicating a disconnect between their self-perceptions and others’ perceptions of them, consistent with the defensive interpersonal style linked to rivalry (Back et al., 2013; Kirk et al., 2022). Thus, narcissistic rivalry may contribute to relational tension and misunderstandings within teams, potentially reinforcing cycles of antagonism and undermining team cohesion. Notably, there was no evidence of other-derogation effects, such as seeing team members as less competent or creative, nor were additional associations with target effects observed. In naturalistic settings, individuals high in rivalry are evaluated by others in relatively neutral terms beyond interpersonal traits (Rauthmann, 2011), which could explain the absence of broader relational effects beyond perceptions of aggression and arrogance.

In summary, our findings illustrate the complex role of narcissism in team contexts. Narcissistic rivalry was negatively associated with team cohesion and, in turn, team performance. In contrast, the contribution of narcissistic admiration was more nuanced, shaping how individuals were perceived by others without notably affecting team-level processes.

## 5. Strengths, Limitations, and Future Directions

The findings have implications for research on personality and teams. Practically, the findings are consistent with research indicating that personality is relevant to team processes and performance (Filipiak & Łubianka, 2020; Tasa et al., 2010; van Vianen & De Dreu, 2001) and that narcissism is worth examining within the team context (Lynch et al., 2021; Xu & Benson, 2024). Methodologically, the study moves beyond traditional self-report and artificial laboratory contexts by using a naturalistic team environment and adopting social relations modelling to capture how narcissistic traits manifest during live, goal-directed teamwork. As such, the findings highlight the value of incorporating round-robin designs when investigating interpersonal phenomena in teams and demonstrate that narcissists are not always cohesive team members.

Despite these strengths, several limitations warrant consideration, chief among them the team size and the familiarity among team members. Teams ranged from four to five members, and 11 teams (47.8%) included participants with some prior acquaintance. As team size and familiarity can influence team processes and outcomes (Gevers et al., 2020; Wheelan, 2009), this design feature may have constrained the generalizability of the findings. Furthermore, the reliance on self-selected participants may have increased the likelihood that some teams included pre-existing relationships, potentially reducing the emergence of conflict during the task. Regardless, the team composition and overall sample size represented a notable improvement over previous escape room research, which has typically involved smaller teams and fewer than 40 participants (Pan et al., 2017).

Another feature of the results was the strong correlations among cohesion, conflict, and performance, as well as the high association between subjective and objective performance. Although these constructs are conceptually distinct, such overlap is not unusual in small-group research, particularly in short, high-pressure tasks where members’ perceptions of group functioning are closely tied to task outcomes (De Dreu & Weingart, 2003). The high convergence between subjective and objective performance likely reflects the salience of the escape room outcome as a shared benchmark for success. While this overlap limits the ability to fully disentangle their effects, it also provides evidence of convergent validity. Future research could employ more differentiated performance metrics or extend the timeframe to capture divergence between subjective impressions and objective results.

Further, the short-term and time-pressured escape room context used may not fully capture the long-term interpersonal influences of narcissistic traits, which may intensify as team interactions develop over time. Although some studies have explored the link between narcissism and team dynamics over time (Carlson & DesJardins, 2015; Lynch et al., 2021), more research is needed to test how narcissism contributes to team processes and performance during longer tasks and across different stages of team development. For instance, narcissists are particularly motivated to seek opportunities for self-enhancement in high-pressure situations (Wallace & Baumeister, 2022) and actively pursue contexts that allow them to demonstrate their abilities (Nevicka & Sedikides, 2021; Sedikides & Campbell, 2017), suggesting that their behaviors and impact on team processes may vary depending on the task type and task duration.

Team composition (e.g., individual differences between members) may also play a role. Research on team creativity identified a curvilinear relation with team-level narcissism, where some narcissism can enhance creativity, but too much becomes detrimental (Goncalo et al., 2010). Follow-up investigations could explore whether narcissism is linked to stage-specific or curvilinear effects on team processes across the lifespan on team tasks. Given that team composition and team structure influence team functioning (Higgs et al., 2005), examining the dispersion of narcissism within teams, as well as the roles individuals occupy, may offer a more complete understanding of how narcissism contributes to team processes and performance.

Another limitation concerns the statistical power of our mediation analyses. Although our models explained substantial variance in performance outcomes (R^2^ = .35–.37), the sample size (*k* = 23 teams; N = 101 participants) was modest for detecting indirect effects. We addressed this by using bootstrapping with 5000 resamples (Hayes, 2022), a widely recommended approach that does not assume normality of the indirect effect and enhances sensitivity in smaller samples. Nevertheless, the limited power means that non-significant findings should be interpreted with caution, as subtle mediation pathways may not have been detectable. Consistent with this, the sensitivity analysis suggests that null team-level effects (e.g., for admiration) may reflect insufficient power, and that smaller but potentially meaningful effects could exist. Replication with larger samples will be important to establish the robustness of these processes.

Although we included peer ratings alongside self-ratings to reduce bias, reliance on self-report measures still introduces potential inaccuracies, particularly for traits linked to self-presentation (Nichols & Maner, 2008). Finally, whereas the naturalistic escape room setting offers ecological validity, it constrains the generalizability of findings to other environments, such as hierarchical teams, cross-functional teams, and operational teams.

## 6. Conclusions

Understanding narcissism within team settings is essential, given the widespread reliance on teams in real-life settings. This study was a step in that direction. Narcissistic rivalry undermined team cohesion and performance, and admiration influenced interpersonal perceptions but not team processes or performance. In addition, relational analyses clarified how narcissistic individuals see themselves and are perceived by others. The findings are generative and point to the value of ecological settings in investigating narcissism.

## Figures and Tables

**Table 1:** Means, Standard Deviations and Reliability Coefficients for all Measures.

Measures	*M*	*SD*	*Skew*	Kurtosis	α	ICC (1)	ICC (2)
**Individual-Level**							
Narcissistic Admiration	4.27	1.26	.31	−.23	.85		
Narcissistic Rivalry	2.66	1.01	.62	.01	.78		
Team Cohesion	6.05	1.33	−.72	.22	.95		
Team Conflict	2.65	1.37	.99	.48	.92		
Impression Management	5.04	1.25	−.28	−.37	.74		
Subjective Team Performance	5.38	1.64	−.35	−.87	.91		
**Team-Level**							
Narcissistic Admiration	4.27	.58	−.86	1.02			
Narcissistic Rivalry	2.66	.49	.64	−.32			
Team Cohesion	6.05	.80	−.54	−.86		.18	.50
Team Conflict	2.65	.86	.55	−.68		.22	.56
Subjective Team Performance	5.38	1.46	−.26	−1.12		.74	.93
Objective Team Performance	1.83	1.12	−.39	−1.26			

*Note.* ICC (1) = proportion of variance in individual scores attributable to team membership; ICC (2) = reliability of the team mean scores on the variable of interest.

**Table 2:** Correlations Among Team-Level Narcissism, Cohesion, Conflict, and Performance Measures.

	1	2	3	4	5	6
1. Narcissistic Admiration	-					
2. Narcissistic Rivalry	.52 ***	-				
3. Impression Management	−.24 *	−.26 **	-			
4. Team Cohesion	−.03	−.22 *	.06	-		
5. Team Conflict	.09	.06	−.07	−.64 ***	-	
6. Subjective Performance	.17	.13	−.07	.54 ***	−.44 ***	-
7. Objective Performance	.31 **	.36 ***	−.13	.37 ***	−.29 ***	.90 ***

*Note*. N = 101. *** *p* < .001. ** *p* < .01. * *p* < .05.

**Table 3:** Summary of Multiple Mediation Models Using PROCESS Model 4 Across Team-Level IVs, Mediators, and Team Performance.

IV	M	DV	Path	*b*	SE	*p*	95% CI
Rivalry		Subjective Team Performance	Total effect (*c*)	.01	.36	.978	[−.72, .74]
			Direct effect (*c*’)	.52	.32	.107	[−.11, 1.15]
	Cohesion		Indirect (Cohesion)	−.48	.22		[−.95, −.12]
	Conflict		Indirect (Conflict)	−.02	.06		[−.20, .09]
			R^2^	.37			
Rivalry		Objective Team Performance	Total effect (*c*)	.49	.26	.065	[−.03, 1.02]
			Direct effect (*c*’)	.82	.25	.001	[.33, 1.31]
	Cohesion		Indirect (Cohesion)	−.32	.15		[−.65, −.08]
	Conflict		Indirect (Conflict)	−.00	.04		[−.10, .05]
			R^2^	.35			
Admiration		Subjective Team Performance	Total effect (*c*)	.34	.29	.246	[−.24, .92]
			Direct effect (*c*’)	.20	.24	.408	[−.28, .69]
	Cohesion		Indirect (Cohesion)	.16	.17		[−.09, .58]
	Conflict		Indirect (Conflict)	−.02	.06		[−.16, .08]
			R^2^	.37			
Admiration		Objective Team Performance	Total effect (*c*)	.34	.21	.11	[−.07, .76]
			Direct effect (*c*’)	.24	.19	.215	[−.14, .62]
	Cohesion		Indirect (Cohesion)	.10	.10		[−.07, .36]
	Conflict		Indirect (Conflict)	−.01	.03		[−.08, .04]
			R^2^	.36			

*Note.* Model (No. 4) controls for gender, impression management, and the other form of narcissism. 5000 bootstrap samples. For the indirect effect tests, significant mediation is evidenced by CIs that do not include zero. Conflict and cohesion were entered as parallel mediators. Unstandardized betas are reported.

**Table 4:** Univariate Analyses of Round Robin Interpersonal Perceptions at Time One and Time Two.

	Perceiver	Target	Relationship (Error)	Perceiver	Target	Relationship (Error)
	Time 1			Time 2		
Confident	.19 (.12) ***	.33 (.18) ***	.48 (.11) ***	.16 (.12) **	.33 (.18) ***	.51 (.12) ***
Supportive	.27 (.15) ***	.12 (.11) *	.61 (.12) ***	.28 (.14) ***	.16 (.12) **	.56 (.13) ***
Arrogant	.47 (.21) ***	.08 (.09) *	.45 (.12) ***	.38 (.23) ***	.11 (.17) *	.51 (.16) ***
Competent	.30 (.13) ***	.10 (.09)	.59 (.11) ***	.40 (.17) ***	.06 (.07)	.54 (.11) ***
Aggressive	.36 (.19) ***	.005 (.09)	.63 (.17) ***	.39 (.19) ***	.13 (.12) *	.48 (.13) ***
Likeable	.29 (.15) **	.06 (.10)	.66 (.15) ***	.28 (.16) ***	.15 (.12) **	.57 (.14) ***
Creative	.21 (.15) **	.16 (.15) **	.63 (.17) ***	.35 (.17) ***	.13 (.11) **	.52 (.12) ***
Trustworthy	.40 (.16) ***	.006 (.06)	.59 (.11) ***	.39 (.16) ***	.06 (.08)	.55 (.11) ***
Empathic	.25 (.15) **	.05 (.10)	.70 (.15) ***	.37 (.18) ***	.20 (.14) **	.43 (.11) ***
Hardworking	.45 (.19) ***	.12 (.09) **	.43 (.10) ***	.32 (.15) ***	.16 (.10) **	.51 (.12) ***
Good Leader	.13 (.18)	.07 (.14)	.80 (.21) ***	.25 (.22) ***	.19 (.20) **	.55 (.19) ***

*Note.* Standardized variance components are reported. The values in brackets are standard error values. Time 1 = Post-Icebreaker; Time 2 = Post-Escape Room. *** *p* < .001. ** *p* < .01. * *p* < .05.

**Table 5:** Assumed Similarity and Self-Other Agreement in Round-Robin Ratings of Interpersonal Attributes.

	Time One		Time Two	
	Assumed Similarity	Self-Other Agreement	Assumed Similarity	Self-Other Agreement
Confident	.13	.26 *	.31 **	.19
Supportive	.22	.19	.52 ***	.20
Arrogant	.41 ***	.10	.53 ***	.16
Competent	.31 **	.08	.40 ***	.32 **
Aggressive	.29 *	.11	.42 ***	.001
Likeable	.48 ***	.27 *	.53 ***	.10
Creative	.23 *	.16	.38 **	.01
Trustworthy	.21	.12	.28 *	−.05
Empathic	.16	.30 **	.31 **	.36 **
Hardworking	.29 *	.19	.29 **	.14
Good Leader	.22	.24 *	.19	.26 *

*Note*. Values are partial correlations between self-ratings and perceiver effects (assumed similarity) and between self-ratings and target effects (self-other agreement), controlling for team membership. Time 1 = Post-Icebreaker; Time 2 = Post-Escape Room. *** *p* < .001. ** *p* < .01. * *p* < .05.

**Table 6:** Partial Correlations Between Narcissism and Interpersonal Measures (Controlling for Team Membership).

Measure		Admiration Time 1	Admiration Time 2	Rivalry Time 1	Rivalry Time 2
Confident	Other	.35 **	.22 *	.05	−.02
	Self	.37 ***	.31 **	−.16	−.16
	Self-Enhancement	.02	.08	−.13	−.009
Supportive	Other	−.001	−.07	−.08	.06
	Self	.17	.25 *	−.17	−.09
	Self-Enhancement	−.06	.19	−.05	−.02
Arrogant	Other	.28 *	.23 *	.04	−.05
	Self	.20	−.04	.21	.24 *
	Self-Enhancement	.19	−.09	.03	.09
Competent	Other	.06	−.08	−.01	.09
	Self	.24 *	.11	−.06	−.09
	Self-Enhancement	.09	.03	−.06	−.17
Aggressive	Other	.11	.13	.22 *	−.07
	Self	.05	−.06	.25 *	.23 *
	Self-Enhancement	.15	−.08	.07	.19
Likeable	Other	.16	−.11	.08	.19
	Self	.36 **	.36 ***	−.05	−.02
	Self-Enhancement	.08	.24 *	−.08	−.28 *
Creative	Other	−.06	−.05	−.10	.04
	Self	.06	.15	−.23 *	−.21
	Self-Enhancement	.07	.04	−.05	−.15
Trustworthy	Other	−.14	−.08	−.08	.12
	Self	.12	.11	−.06	−.23 *
	Self-Enhancement	.00	.01	.08	−.14
Empathic	Other	−.16	−.35 **	−.15	−.01
	Self	−.14	−.04	−.09	−.14
	Self-Enhancement	−.14	.15	.08	−.001
Hardworking	Other	−.24 *	−.10	−.25 *	.02
	Self	.06	−.04	−.25 *	−.15
	Self-Enhancement	.08	−.03	−.004	−.11
Leader	Other	.29 **	.15	−.08	.17
	Self	.28 *	.24 *	−.07	−.03
	Self-Enhancement	.08	.04	.04	−.03

*Note.* Partial correlations are reported controlling for team membership. “Other” = target effects (team ratings), “Self” = self-perceptions. Time 1 = Post-Icebreaker; Time 2 = Post-Escape Room. *** *p* < .001. ** *p* < .01. * *p* < .05.

## Data Availability

Data for this study can be found here: https://osf.io/gyvft/?view_only=6a5264f7816b4bf383e2113ca8c782da (accessed on 1 August 2025).

## Notes

1 Of teams, 12 (52.17%) were composed entirely of strangers, eight (34.78%) included two participants who knew each other before the study, and three (13.05%) had at least two participants who were acquainted before the study.

2 To assess whether familiarity (i.e., the proportion of team members knowing at least one other member) influenced team-level cohesion and conflict, additional analyses including familiarity as a covariate were run. Familiarity yielded no significant effects and did not change the overall pattern of results.
